# Using a mobile application to reduce potentially inappropriate prescribing for older Brazilian adults in primary care: a triple-blind randomised clinical trial

**DOI:** 10.1186/s12877-023-04645-z

**Published:** 2024-01-08

**Authors:** Welma Wildes Amorim, Luiz Carlos Passos, Romana Santos Gama, Renato Morais Souza, Pablo Moura Santos, Jéssica Caline Macedo, Hévila Maciel Queiroga, Larissa Gusmão Nunes, Lavínia Mendonça Fraga, Brunna Santos Oliveira, Lucas Teixeira Graia, Marcio Galvão Oliveira

**Affiliations:** 1 Department of Health, State University of Southwest Bahia, Vitória da Conquista Campus, Vitória da Conquista, Bahia, Brazil; 2 Department of Internal Medicine, Postgraduate Program in Medicine and Health, Federal University of Bahia, Salvador, Bahia Brazil; 3 Postgraduate Program in Pharmaceutical Services and Policies, Federal University of Bahia, Salvador, Bahia Brazil; 4 Federal University of Bahia, Vitória da Conquista, Uberlândia, Bahia, Brazil; 5 Multidisciplinary Institute in Health– Anísio Teixeira Campus, Federal University of Bahia, Vitória da Conquista, Bahia, Brazil

**Keywords:** Aged, Potentially inappropriate medications, Prescription drugs, Primary health care, Deprescribing

## Abstract

**Backgound:**

Potentially inappropriate prescribing (PIP) has been evaluated in several countries, and several strategies have been devised for deprescribing drugs in older adults. The aim of this study was to evaluate the efficacy of a mobile application in reducing PIP for older adults in primary care facilities in Brazil.

**Methods:**

This randomised, triple-blind, parallel-group trial was conducted in 22 public primary care facilities in Brazil. During the intervention phase, the general practitioners (GPs) were randomly allocated to the intervention (MPI Brasil app provides information about PIP, therapeutic alternatives and deprescribing) or control (MedSUS app provides general information about medications) group. All GPs were trained on the Clinical Decision-Making Process and how to access an Evidence-Based Health website. The GPs received an Android tablet with an installed mobile application depending on their allocated group, which they used when caring for older patients over at least 3 months. At the end of this period, a sample of older patients aged ≥ 60 years who had been awaiting medical consultation by the participating GPs were interviewed and their prescriptions analysed. The primary outcome was the frequency of PIP in and between the groups.

**Results:**

Among 53 GPs who were administered the baseline survey, 14 were included in the clinical trial. At baseline, 146 prescriptions were analysed: the PIP overall was 37.7% (55/146), in the intervention group was 40.6% (28/69), and in the control group was 35.1% (27/77). After the intervention, 284 prescriptions were analysed: the PIP overall was 31.7% (90/284), in the intervention group was 32.2% (46/143), and in the control group was 31.2% (44/141) (RR: 1.16; 95% CI, 0.76–1.76). In the within-group analysis, the PIP reduced from before to after the intervention in both groups—more significantly in the intervention than in the control group (*p* < 0.001). In the stratified analysis of PIP frequency by GPs, there was a relative risk reduction in 86% (6/7) of GPs in the intervention group compared to 71% (5/7) in the control group.

**Conclusion:**

We found that the MPI Brasil app effectively reduced PIP, suggesting that it may be useful to incorporate this tool into clinical practice.

**Trial registration:**

The study was registered at ClinicalTrials.gov (NCT02918643). First registration on 22/09/2016.

## Background

Inappropriate prescribing has been defined as prescribing medications with more potential risk than benefit, or not complying with accepted medical standards [1]. In older adults, potentially inappropriate prescribing (PIP) has been associated with hospitalisations [2, 3], emergency room visits, adverse drug events, functional decline, and mortality [3]. PIP can be measured using explicit criteria (criterion-based) [4, 5], and interventions using explicit criterion-based tools are an essential component of strategies to reduce PIP [6, 7].

Information and communication technologies have been used to improve the appropriateness of medication prescribing [8] as they allow rapid access to explicit criteria during patient care. Several studies have evaluated the positive and negative impacts of informative/educational mobile applications that aim to improve the quality of care in the community and in-hospital settings [9–11]. Mobile apps can instantly connect clinicians to a wealth of information, with the advantages of being portable, easy to use, customizable, low cost and accessible at the point of care [10].

In a study conducted in Brazil among community-dwelling older adults, 34.5% used at least one potentially inappropriate medication (PIM) and among the associated factors, the use of medication prescribed by a physician is notable [12]. In another study conducted in Brazilian primary care, 45.3% of the patients received at least one PIP after medical consultations; 86.8% of physicians supplied at least one PIP; and the factors associated with inappropriate prescribing to older patients included the number of primary care patients attended to by a general practitioner (GP) and medical practice experience under 10 years [13]. Therefore, tools enabling access to reliable information may prevent PIP and improve deprescribing initiatives. Accurate information can facilitate the benefit–risk assessment of a particular treatment for a patient and evaluation of the patient’s cultural, social, and economic preferences, especially in the older population [14]. Therefore, this study aimed to evaluate the efficacy of an informative mobile application in reducing PIP for older adults in primary care facilities in Brazil.

## Methods

### Study design

This study was a randomised, triple-blind, active-controlled clinical trial with two parallel groups.

### Participants

**Fig. 1:**
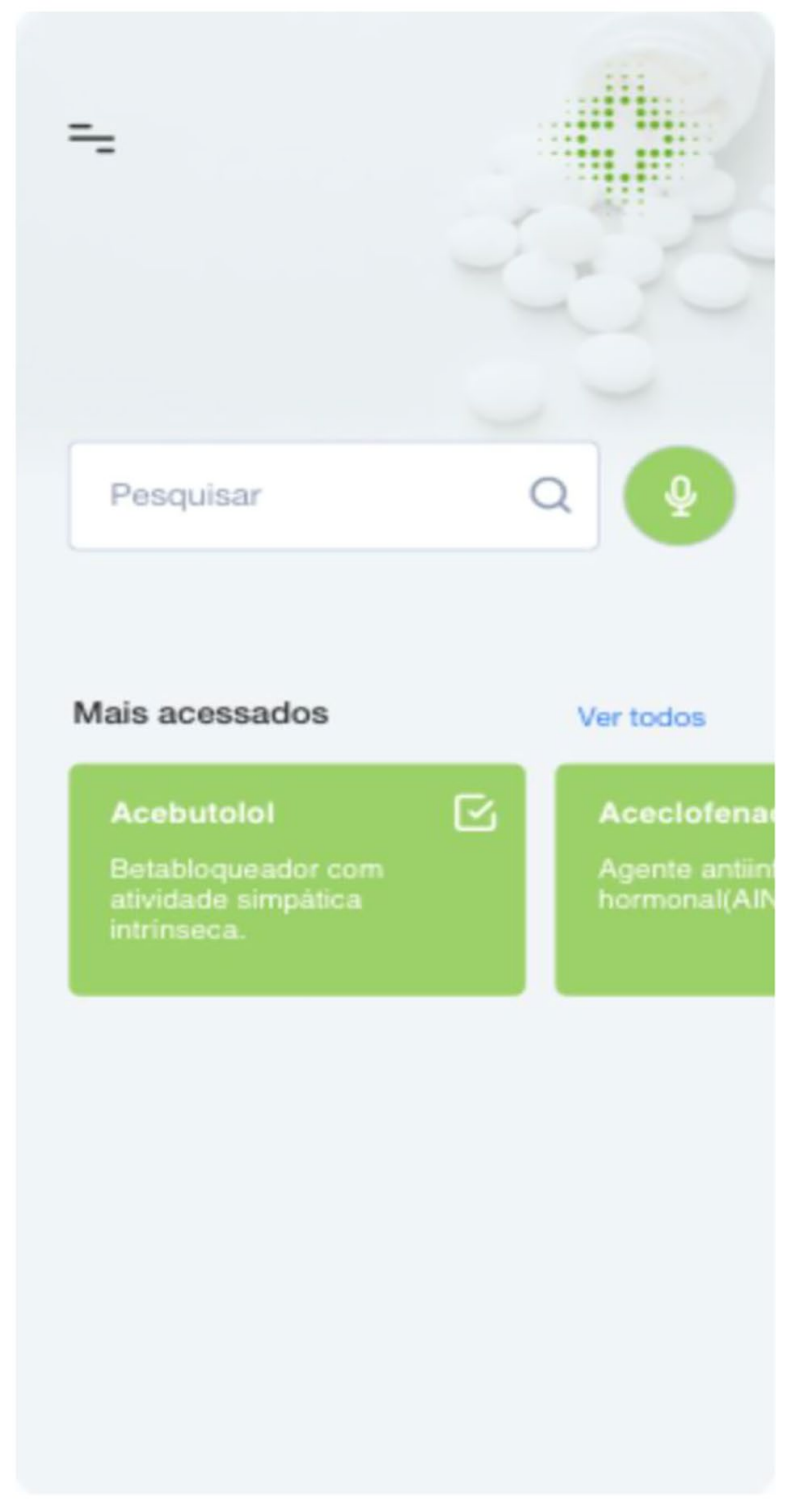
MPI Brasil search bar

The prescriptions of 53 GPs who treated older adults at 22 public primary care facilities in Vitória da Conquista, a municipality in north-eastern Brazil, were evaluated. At baseline, a cross-sectional study was conducted [13] to identify the occurrence of PIP among 146 GP prescriptions and select those eligible for the intervention phase (Fig. 1).

### Inclusion criteria

GPs working in the study facilities, who had issued at least five prescriptions and with at least one PIP incident at baseline were included.

### Exclusion criteria

GPs who were unavailable to participate in training during the intervention phase and those who stopped working at the study facilities during the data gathering period were excluded.

### Interventions

We developed an Android informative application based on the Brazilian Consensus on Potentially Inappropriate Medication for Older People. This consensus was developed and validated by Brazilian researchers and contains information about PIM used in Brazil for older adults [15]. The application, called ‘MPI Brasil’, includes the rationale for a drug to be considered as a PIM, the classification—whether classified as a PIM regardless of the clinical condition, depending on the clinical condition, or both—and the exceptional situations in which the medication would not be considered a PIM. It also contains information on the safest therapeutic alternatives, deprescribing, and how to monitor whether using a PIM is essential. The app MPI Brasil has a search field. To access the information, the user must type a few letters or the full name of the medication for which information is being sought (Fig. 1) [16].

**Fig. 2:**
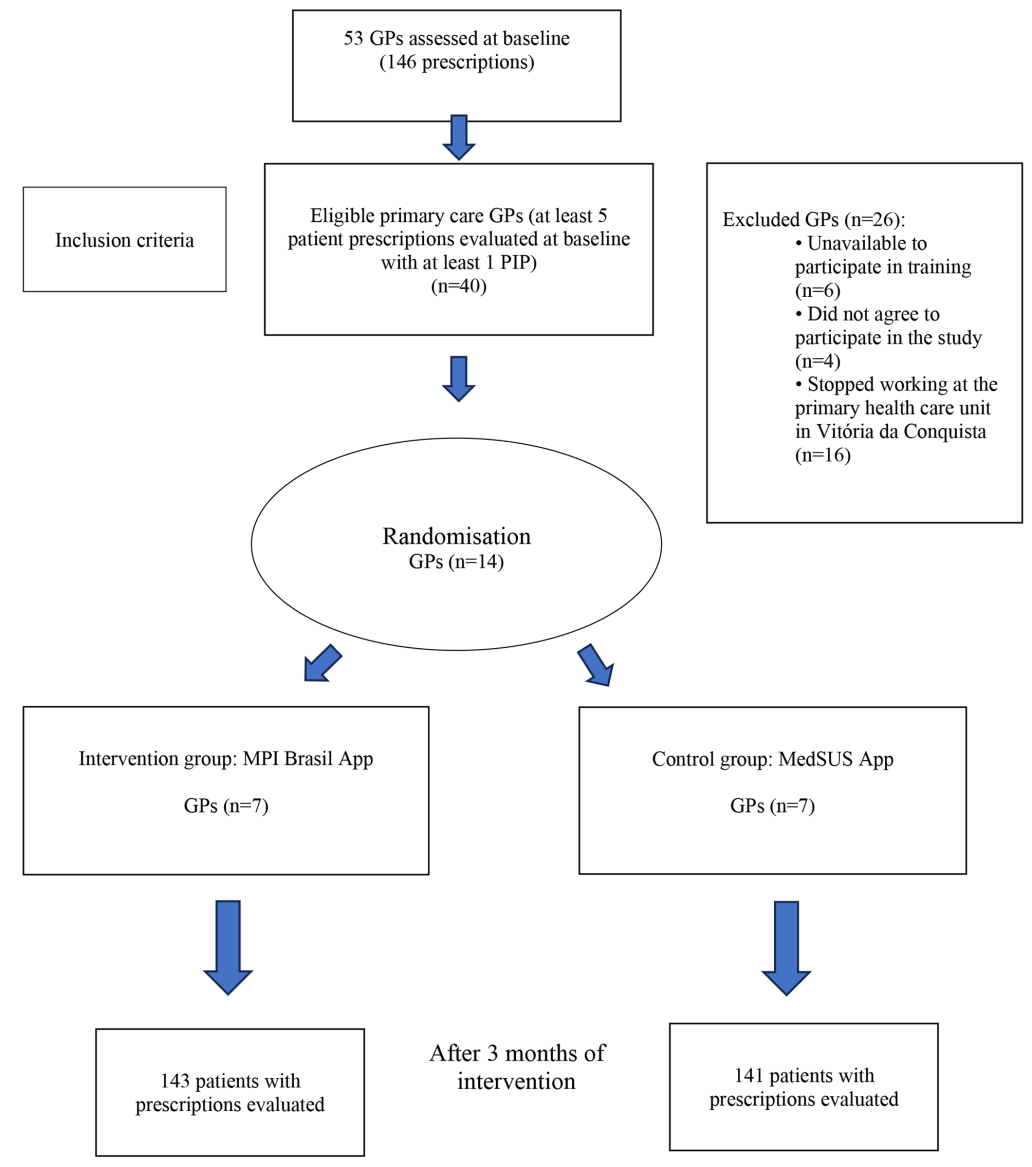
Participant flowchart GP, general practitioner; PIP, potentially inappropriate prescribing

During the intervention phase, 14 eligible GPs from a different health facility were invited to participate in the study and were randomly allocated to the intervention or control group (Fig. 2).

All GPs were trained on the Clinical Decision-Making Process and how to access the Evidence-Based Health website of the Brazilian Ministry of Health. The training was carried out with each GP individually in theoretical classes. After the theoretical guidance, each GP carried out information search exercises following the methodology applied in the training. The GPs were tutored during the period when they had questions and difficulties. The Evidence-Based Health website of the Brazilian Ministry of Health offers rapid, direct access to the information needed to make diagnostic and clinical decisions, assists in research, and provides medical education through various databases, such as Access Medicine, Dynamed, and Micromedex [17].

All GPs received an Android tablet to access the Evidence-Based Health website. The MPI Brasil app [16] was only installed on the tablets of the intervention group. In the control group, we installed an application called MedSUS, made available by the Brazilian Ministry of Health, which provides information about the medications on the Brazilian list of essential medicines [18]. During the intervention period, the MPI Brasil app [16] was removed from Google Play to avoid access by the control group. The GPs used the tablets when caring for older patients over at least 3 months. In both groups, GPs were advised to access the applications during consultations to improve the standard of prescriptions for older patients. At the end of this period, a sample of patients aged ≥ 60 years was interviewed in the waiting rooms of the participating GPs and their prescriptions were analysed following the same baseline procedures: in terms of the composition of their active ingredients. Prescriptions were considered to be PIMs based on the Brazilian consensus on potentially inappropriate medications for elderly people, with regard to rationale, clinical condition and exceptions [12]. We administered a multidimensional questionnaire using the KoBoToolbox application (KoBoToolbox®, Cambridge, MA, USA) before and after the GPs attended to the patients at the study facilities [12]. The duration of the consultation as well as the prescription and chart data were recorded for subsequent evaluation. Data were collected between December 2017 and March 2019.

### Outcomes

The primary outcome was the frequency of PIP based on the Brazilian Consensus on Potentially Inappropriate Medication for Older People [15], with regard to rationale, clinical condition, and exceptions based on the data collected from the patients.

### Sample size

For the sample size calculation, we assumed a 29% expected frequency of PIP before the intervention and a 14% expected difference between the intervention and control groups after the intervention. In the two-tailed hypothesis test, a significance level of 5% and power of 80% were used. A sample size of 284 was determined, which was proportionally divided by the number of GPs in the intervention and control groups (Fig. 1).

### Randomisation

Randomisation was performed by a non-blinded, independent member of the research team who was not involved in the outcome assessment. After verifying the eligibility criteria, 14 GPs were randomly assigned to the intervention or control group at a 1:1 ratio using the permuted block technique, and stratified by sex and time since graduation in years. The generated random sequence was based on a previous classification of the GPs based on sex and time since graduation strata (≤ 10 years or > 10 years). After stratification, GPs were randomised using a sequence of blocks with random variation (four participants per block).

### Implementation

The result of randomisation was shared only with the investigator who was responsible for inviting GPs to participate in the study, enrolling them in the research, providing them with tablets, and training each participant individually according to the protocol of the group to which they were allocated.

### Blinding

GPs were blinded to their assignment to the intervention or control group and each participating GP was trained individually in the intervention phase. Other members of the health teams of the study facilities and the data collection team that interviewed patients in the post-intervention phase were also blinded. During the data analysis phase, the team member who conducted the PIP assessment according to the criteria adopted in this study was also blinded.

### Statistical analysis

Data were analysed using SPSS Statistics for Windows, Version 25.0 (IBM Corp, Armonk, NY, USA). Simple frequency analysis of the categorical variables of interest was performed using the χ^2^ and McNemar tests to assess—between and within the groups, respectively—the difference between the PIP frequency before and after the intervention. In the case of continuous variables, the t-test was used to compare means, if all statistical assumptions were met; else, the Mann–Whitney U test was used. Differences with *p* < 0.05 were considered significant.

The relative risk (RR) and 95% confidence interval (CI) and the RR reduction (RRR), taking the PIP frequency before and after the intervention into account, were evaluated within each group and the two groups were compared.

### Ethics

This study was based on the principles of the Declaration of Helsinki, and approved by the Multidisciplinary Institute in Health Research Ethics Committee (approval number 378.198). All GPs and older patients provided written informed consent.

## Results

Eight of the 14 participating GPs (57%) were women, with a mean age of 45.9 years. Ten of the participating GPs (71%) had graduated more than 10 years prior and four (29%) had completed residency training (Table 1).

**Table 1:** Characteristics of GPs included in the clinical trial

GP characteristic	General (*N* = 14)	Intervention group (*N* = 7)	Control group (*N* = 7)
Sex, n (%)			
Male	6 (43)	3 (43)	3 (43)
Female	8 (57)	4 (57)	4 (57)
Age (years), mean (95% CI)	45.9 (37.4–54.5)	44.7 (29.0–60.4)	47.1 (34.7–59.6)
Time since graduation, n (%)			
≤ 10 years	4 (29)	2 (29)	2 (29)
> 10 years	10 (71)	5 (71)	5 (71)
Medical residency, n (%)			
Yes	4 (29)	2 (29)	2 (29)
No	10 (71)	5 (71)	5 (71)

CI, confidence interval; GP, general practitioner

At baseline, 146 GP prescriptions were evaluated, among which 55 (37.7%) were classified as PIP, being more frequent in the intervention (40.6%) than in the control group (35.1%). After the intervention, the overall PIP rate was 31.7% (90/284); however, the between-group analysis revealed that the difference in PIP frequency between the intervention (32.2%) and control group (31.2%) was not significant (*p* = 0.862). The within-group analysis, before and after the intervention, revealed that the PIP frequency reduced in both groups, but more significantly in the intervention group (*p* < 0.001) (Table 2). In the stratified analysis of PIP frequency by GP, a RRR was seen in 86% (6/7) of GPs in the intervention group compared with 71% (5/7) in the control group (Table 3).

**Table 2:** Comparative analysis of the frequency of PIP before and after intervention in the control and intervention groups

Study phase	General	Intervention group	Control group	RR
**Pre-intervention**				
PIP, % (n/N)	37.7 (55/146)	40.6 (28/69)	35.1 (27/77)	1.16 (0.76–1.76)
**Post-intervention**				
PIP, % (n/N)	31.7 (90/284)*	32.2 (46/143)*	31.2 (44/141)*	1.03 (0.73–1.45)

PIP, potentially inappropriate prescribing; RR, relative risk

*p = 0.862 in within-group analysis (χ² test) and *p* < 0.001 in between-group analysis (McNemar test)

**Table 3:** PIP frequency in the pre-and post-intervention phases by GP

Group and GP	Pre-intervention phase					Post-intervention phase				RRR (%)
	PIP No (n)	PIP Yes (n)	PIP (%)	RR PIP		PIP No (n)	PIP Yes (n)	PIP (%)	RR PIP	
**Control**										
C1	9	2	18	0.22		23	3	12	0.13	33
C2	2	3	60	1.50		17	3	15	0.18	75
C3	10	2	17	0.20		7	10	59	1.43	−247
C4	7	4	36	0.57		14	4	22	0.29	39
C5	10	5	33	0.50		11	8	42	0.73	−27
C6	5	7	58	1.40		10	10	50	1.00	14
C7	7	4	36	0.57		15	6	29	0.40	19
**Intervention**										
I1	2	4	67	2.00		14	6	30	0.43	55
I2	7	3	30	0.43		20	6	23	0.30	23
I3	6	4	40	0.67		19	9	32	0.47	20
I4	8	6	43	0.75		4	0	0	0.00	100
I5	9	3	25	0.33		8	9	53	1.13	−112
I6	4	3	43	0.75		15	8	35	0.53	19
I7	5	5	50	1.00		17	8	32	0.47	36

C, control; I, intervention; PIP, potentially inappropriate prescribing; RR, relative risk; RRR, relative risk reduction

After the intervention, the ten most prescribed potentially inappropriate medications coincided between the intervention and control groups, except that in the intervention group there was a prescription for Nifedipine (risk of hypotension and myocardial ischemia), Benzodiazepines (risk of delirium, falls, fractures) and 1st generation antipsychotics. In the control group, there was a prescription for H2 receptor antagonist (risk of worsening cognitive impairment), anticonvulsants (syncope, impairment of psychomotor function, risk of falls), except in seizures and 1st generation antihistamines (highly anticholinergic agent) (Table 4).

**Table 4:** Top ten medications in PIP in intervention and control group

Intervention Group	PIP (n)	Control Group	PIP (n)
NSAID: ibuprofen, meloxicam and others	10	Long-term use of corticosteroids (as monotherapy for osteoarthritis or rheumatoid arthritis): betamethasone, prednisone	10
Long-acting sulfonylurea: glyburide	6	NSAID: ibuprofen, meloxicam and others	8
Long-term PPI: omeprazole, dexlansoprazole	6	Long-acting sulfonylurea: glyburide	7
Non-cardioselective beta-blocker if COPD: propranolol, carvedilol, metoprolol	4	H2 receptor antagonist (risk of worsening cognitive impairment): ranitidine	5
Tricyclic antidepressant (highly anticholinergic agent): amitriptyline	4	Furosemide (first line for hypertension)	4
Nifedipine (risk of hypotension and myocardial ischemia)	3	Non-cardioselective beta-blocker if COPD: propranolol	4
Furosemide (first line for hypertension)	3	Long-term PPI: omeprazole	3
Long-term use of corticosteroids (as monotherapy for osteoarthritis or rheumatoid arthritis): betamethasone	3	Anticonvulsants (syncope, impairment of psychomotor function, risk of falls). Avoid except in seizures: carbamazepine, topiramate, phenytoin	3
Benzodiazepines (risk of delirium, falls, fractures): clonazepam, midazolam	3	1st generation antihistamines (highly anticholinergic agent): dexchlorpheniramine, dimenhydrinate)	3
1st generation antipsychotics: haloperidol, chlorpromazine, thioridazine	3	Tricyclic antidepressant (highly anticholinergic agent): amitriptyline	3

## Discussion

This real-world clinical trial conducted in the Brazilian public health system evaluated the efficacy of a mobile application in reducing PIP for older adults in primary care facilities. At first glance, in the between-group analysis, there was no statistically significant difference in the RR of PIP between the intervention and control group. However, per the within-group analysis, there was a significant reduction in the frequency of PIP in both groups of GPs, with a larger reduction in the intervention group. Furthermore, in the stratified analysis of PIP frequency by GP, more GPs in the intervention group (MPI Brasil app) than in the control group (MedSUS app) showed an RRR.

This randomized controlled trial effectively employed a combination of strategies aimed at PIP reduction in primary care through educational interventions via mobile computerized systems to support clinical decision-making. The training on the Clinical Decision-Making Process and how to access the Evidence-Based Health website of the Brazilian Ministry of Health [17] was conducted in both groups and empowered the GPs to obtain the best available evidence to improve patient outcomes, leading to higher-quality health care. The MedSUS app [18] used by the GPs in the control group was not a placebo and contended with the MPI Brasil app [16] to reduce PIP. The MedSUS app of the Brazilian Ministry of Health was developed to promote the rational prescription of medicines by providing scientific information of drugs from the Brazilian list of essential medicines in addition to therapeutic guidelines and clinical protocols recommended by the Brazilian Ministry of Health [18].

Meanwhile, the MPI Brasil app was developed specifically to provide access to compiled information about PIMs to promote safer prescribing for older adults. This mobile application not only informs users which medications are considered potentially inappropriate and the rationale behind it, but also contains therapeutic alternatives; gradual deprescribing guidelines, in case the medication cannot be withdrawn abruptly; and monitoring guidelines, when the prescription or maintenance of inappropriate medication is considered necessary, such as in cases of exception or lack of access to safer alternatives [16].

However, various functionalities of the MPI Brasil app [16] could not be measured as part of the outcome of this study, such as deprescribing and monitoring guidelines. Furthermore, despite the decreased frequency of PIP in both groups, some PIMs, such as benzodiazepines, tricyclic antidepressants, and proton pump inhibitors, cannot be terminated abruptly and must be deprescribed gradually [15]. Such medications may have been considered as PIP in our analysis, and this may have led to the impact of the intervention being underestimated.

The intervention was successful despite some barriers that may have influenced the therapeutic choices of GPs, such as the unavailability of safer medications for older patients on the list of essential medications subsidized by the Brazilian public health system [19]. The sample population comprised older adults with a low income, and the therapeutic options available to primary care physicians were often limited to medications available at municipal public pharmacies.

Among the 10 PIPs most prescribed by GPs in the control and intervention groups, it can be noted that there were coincidences in most cases. Almost all potentially inappropriate medications are contained on the list of Brazilian essential medications [19]. The differences in each group can be explained by the clinical conditions of the patients allocated to groups.

Several studies have used online education strategies [20, 21], clinical decision support systems to help health professionals at the time of prescription or during the review of medications in clinical practice [22, 23], and combined interventions [24, 25]. Generally, these clinical decision support systems have been incorporated in electronic medical records, although few studies have used mobile applications as a strategy to reduce PIP [24]. However, this platform is used for other health issues [26, 27].

Opportunities: The choice of a mobile application to reduce PIP is a good strategy because it allows instant access to information and is not limited by the health professional’s location. For example, it can be used at the patient’s bedside. Usually, physicians constantly have access to their phones, in contrast to pocket guides, desktop computers, and reference handbooks. Moreover, the information on a mobile application can be accessed more frequently and from any location, making it easy to update data remotely without needing to issue new physical documents. Given that the MPI Brasil app [16] can transmit information efficiently, PIM information can be more conveniently and rapidly available. Despite the criteria being explicit, retrieving this information from publications—such as the Beers criteria [6], STOPP criteria [28], and the Brazilian Consensus on Potentially Inappropriate Medication for Older People [15]—requires interpretation which may lead to varying interpretations, both in clinical practice and research.

Limitations: First, we used convenience sampling at baseline and after the intervention. The random selection of older adults who would undergo a medical consultation at a relevant municipal primary care unit was not conducted given that, at the time of data collection, no database of all older adults assisted at the health units existed, nor would it have been possible to predict which older adults would have a medical consultation during the data collection period. Second, among the 53 GPs assessed using the baseline survey, only 14 were included in the trial. Many stopped working at the facilities included in the study after the baseline survey. In addition, the sample size calculation did not consider the design effect (clustering of prescribing habits by GP); therefore, the study had insufficient power to show a real difference between the groups. Finally, as this was a real-life study, verifying GPs’ adherence to the use of applications at each consultation was impossible.

The future perspective is to determine strategies for the MPI Brasil app [16] to remain freely available to healthcare professionals who care for older people, thereby ensuring the sustainability of the application and safer prescription for these patients.

## Conclusions

In conclusion, in the within-group and stratified analyses, this trial showed a significantly reduced PIP frequency after both interventions, particularly after the use of the MPI Brasil application by GPs. Nevertheless, there was no significant difference in PIP frequency between the groups after the intervention in the between-group analysis. Choosing the most appropriate medication for each patient to achieve the desired therapeutic results is challenging for health professionals, especially in the care of older patients, an age group in which PIP has become a global problem. The efficacy data of the MPI Brasil app in reducing PIP points to the possibility of incorporating this tool into clinical practice, especially in primary care settings.

## Data Availability

Data that are generated or analyzed during this study are not publicly available to preserve the anonymity of participants, but are available from the corresponding author upon reasonable request.

## Abbreviations

App: Application
GPs: General Practioners
PIM: Potentially Inappropriate Medication
PIP: Potentially Inappropriate Presccribing
